# Impaired Ciliary Beat Frequency and Ciliogenesis Alteration during Airway Epithelial Cell Differentiation in COPD

**DOI:** 10.3390/diagnostics11091579

**Published:** 2021-08-31

**Authors:** Julien Ancel, Randa Belgacemi, Zania Diabasana, Jeanne-Marie Perotin, Arnaud Bonnomet, Maxime Dewolf, Claire Launois, Pauline Mulette, Gaëtan Deslée, Myriam Polette, Valérian Dormoy

**Affiliations:** 1 Inserm UMR-S1250, P3Cell, Université de Reims Champagne Ardenne, SFR CAP-SANTE, 51092 Reims, France; jancel@chu-reims.fr (J.A.); randa.belgacemi@lundquist.org (R.B.); zania.diabasana@inserm.fr (Z.D.); jmperotin-collard@chu-reims.fr (J.-M.P.); arnaud.bonnomet@univ-reims.fr (A.B.); pmulette@chu-reims.fr (P.M.); gdeslee@chu-reims.fr (G.D.); myriam.polette@univ-reims.fr (M.P.); 2 Department of Respiratory Diseases, Centre Hospitalier Universitaire de Reims, Hôpital Maison Blanche, 51092 Reims, France; mdewolf@chu-reims.fr (M.D.); claunois@chu-reims.fr (C.L.); 3 Platform of Cellular and Tissular Imaging (PICT), Université de Reims Champagne Ardenne, 51097 Reims, France; 4 Department of Biopathology, Centre Hospitalier Universitaire de Reims, Hôpital Maison Blanche, 51092 Reims, France

**Keywords:** chronic obstructive pulmonary disease, cilia, airway epithelial cells, CBF, CiliOPD

## Abstract

Chronic obstructive pulmonary disease (COPD) is a frequent respiratory disease. However, its pathophysiology remains partially elucidated. Epithelial remodeling including alteration of the cilium is a major hallmark of COPD, but specific assessments of the cilium have been rarely investigated as a diagnostic tool in COPD. Here we explore the dysregulation of the ciliary function (ciliary beat frequency (CBF)) and differentiation (multiciliated cells formation in air-liquid interface cultures) of bronchial epithelial cells from COPD (*n* = 17) and non-COPD patients (*n* = 15). CBF was decreased by 30% in COPD (11.15 +/− 3.37 Hz vs. 7.89 +/− 3.39 Hz, *p* = 0.037). Ciliary differentiation was altered during airway epithelial cell differentiation from COPD patients. While the number of multiciliated cells decreased (*p* < 0.005), the number of primary ciliated cells increased (*p* < 0.05) and primary cilia were shorter (*p* < 0.05). Altogether, we demonstrate that COPD can be considered as a ciliopathy through both primary non-motile cilia modifications (related to airway epithelial cell repair and remodeling) and motile cilia function impairment (associated with decrease sputum clearance and clinical respiratory symptoms). These observations encourage considering cilia-associated features in the complex COPD physiopathology and highlight the potential of cilia-derived biomarkers for diagnosis.

## 1. Introduction

Chronic obstructive pulmonary disease (COPD) is a common respiratory disease characterized by persistent respiratory symptoms and airflow limitation [1], mainly caused by tobacco smoke and pollutants exposure. While its pathogenesis remains unclear, airway epithelial remodeling appears as a hallmark of COPD [2]. Respiratory symptoms including cough, dyspnea, sputum, and chronic bronchitis are frequent. Combined with airway inflammation [3], mucociliary clearance dysfunction and more especially ciliary epithelial airway impairment may play a key role [4].

Previous studies restricted to nasal brushes demonstrated a decreased ciliary beat frequency (CBF) in COPD patients [5,6,7]. In vitro studies on air-liquid interface (ALI) epithelia from non-COPD and COPD patients did not identify alteration of CBF [8]. We previously explored ciliary dysfunction in COPD regarding airway epithelium differentiation from bronchial cells. We observed that epithelial differentiation was altered in COPD patients, resulting from the dysregulation of the sonic hedgehog signaling [9,10], also supported by non-motile primary cilia (PC) abnormalities [11]. Nonetheless, we did not assess the functionality of the cilia and we only characterized the alteration of ciliogenesis during differentiation on airway epithelial cells isolated from nasal polyp samples. In addition, we recently evidenced via bioinformatics an abnormal cilia-associated genomic signature in COPD patients suggesting a COPD endotype exhibiting ciliopathy features that we named CiliOPD [12]. Here, we hypothesized that the homeostasis of motile cilia function (explored by ciliary beat frequency) and bronchial airway epithelial ciliated cell differentiation are both orchestrated by primary ciliogenesis. Their alterations in COPD patients may originate from dysregulation of primary cilia, an organelle often neglected as a diagnostic tool in respiratory diseases except in the context of primary ciliary dyskinesia [13].

## 2. Materials and Methods

### 2.1. Study Population

COPD and non-COPD control subjects were prospectively recruited from the Department of pulmonary medicine, University Hospital of Reims (France). Non-COPD controls were subjects with no diagnosis of chronic respiratory disease. COPD patients were enrolled based on clinical and functional assessments with a forced expiratory volume in 1 s (FEV_1_)/forced vital capacity (FVC) < 0.7 after bronchodilation. At inclusion, all patients were stable with no acute exacerbation of COPD for 4 weeks. Patients with asthma, cystic fibrosis, tuberculosis, cancer, or other chronic respiratory disease were excluded. Patient characteristics, including demographic data, medical history, treatments, respiratory symptoms, and pulmonary function tests (PFT), were collected. Subjects who had ceased smoking for more than 6 months were considered to be ex-smokers. The severity of COPD was determined according to the spirometric classification (GOLD 1: FEV1 ≥ 80% predicted, GOLD 2: 50% ≤ FEV1 < 80% predicted, GOLD 3: 30% ≤ FEV1 < 50% predicted, GOLD 4: FEV1 < 30% predicted). Frequent exacerbations were defined as at least two exacerbations in the past 12 months [14,15]. All subjects provided written informed consent to the study (Research and Innovation in Chronic Inflammatory Respiratory Diseases-RINNOPARI, NCT02924818). All subjects underwent fiberoptic bronchoscopy with bronchial brushing under routine clinical conditions according to international guidelines [16].

### 2.2. Flexible Fiberoptic Bronchoscopy Procedure

Fiberoptic bronchoscopies were performed under local anesthesia with routine clinical conditions according to international guidelines [16]. Briefly, with monitoring of oxygen saturation, cavum, larynx, vocals cords, and trachea were successively anesthetized. After a careful evaluation of macroscopic endo-bronchial lesions, bronchial brushes were realized in the right lower lobe (4–5th order division). Three bronchial brushes per patient were collected.

### 2.3. Human Primary Airway Epithelial Cell Cultures

Fresh airway epithelial cells (AEC) obtained from bronchial brushings (right lower lobe) were suspended within 30 min in RPMI (1% penicillin/streptomycin+ 10% BSA) before centrifugation (12,500 rpm twice). The cell pellet was dissociated in 1 mL of Trypsin-Versene^®^ (Sigma-Aldrich, Saint Quentin Falavier, France), centrifuged (12,500 rpm twice), and counted with ADAM (NanoEnTek, ThermoFisher Scientific, Waltham, MA, USA) according to NanoEnTek instructions. Total of 200,000 cells were seeded on 12-well plates containing 0.4 µm Polyester Membrane Transwell-Clear Permeable Supports (Cat. No. 3460, Corning, Fisher Scientific; 4 × 10^6^ pores/cm^2^) coated with 0.3 mg/mL collagen type IV from the human placenta (Sigma-Aldrich, Saint Quentin Falavier, France) to establish ALI cultures (passage 0) as described by us and others [10,17,18,19,20,21,22]. PneumaCult-EX (PnC-Ex) media (StemCell, Saint-Egrève, France) was used for initial proliferation in apical and basal chambers. Upon reaching cell confluency after 3 to 5 cell divisions, the apical medium was removed and PneumaCult-ALI (PnC-ALI, StemCell) medium was used in the basal chamber. The culture medium was changed three times a week and cells were kept in incubators at 37 °C, 5% CO2. Cells and supernatants were collected every 7 days for 35 days to generate kinetic analysis. The quantity of cell divisions during the course of the cell culture is 5 to 7 and this is homogenous between biological samples.

### 2.4. Immunofluorescence Staining

Methanol-fixed AEC from ALI cultures were rehydrated by decreasing methanol concentration before post-fixation with acetone. Cells were then blocked with 10% BSA in PBS for 2 h at room temperature and incubated with the primary antibody Arl13b (17711-1-AP, Proteintech, Manchester, UK) to detect cilia as previously described [23,24,25,26] for one night at 4 °C in 3% BSA in PBS. Cells were washed with PBS and incubated with the appropriate secondary antibodies in PBS for 2 h at room temperature. DNA was stained with DAPI during incubation with the secondary antibodies. Clarification of cells was achieved by a glycerol gradient (25%/50%/75%) before mounting the slides. Micrographs were acquired on a Zeiss AxioImager microscope (20× Ph and 63×) with ZEN software (version 8.1, 2012, Zeiss, Marly le Roi, France) and processed with ImageJ (National Institutes of Health, version 2.2.0, Bethesda, MD, USA) for analysis. We routinely control the specificity of the cilia labelling with co-staining and we perform negative and positive controls for each set of experiment [11,27]. The density of multiciliated cells (MCC) was evaluated with the average pixel density of the fluorescence associated to cilia staining as previously described [10]. The percentage of primary ciliated cells and the average cilia length were automatically quantified with CiliaQ as previously described [28].

### 2.5. CBF Analysis

Within 30 min after collection, 200,000 fresh cells were seeded on non-coated µ-Dish 35 mm dishes (Ibidi, 81156) in 400 µL of RPMI (1% penicillin/streptomycin+ 10% BSA) and observed with a video microscope (Axio Observer Z1, Zeiss). At least five acquisitions were acquired by sample (40×- 500 images by 20 ms of exposition). CBF analysis was then performed using CiliaFA as described previously [29] (Supplemental Figure S1).

### 2.6. PCR Analysis

Total RNA from at least 1,000,000 frozen AEC was isolated by High Pure RNA isolation kit (11828665001, Roche Diagnostics, Basel, Switzerland) and quality was assessed with a Nanodrop^TM^ 2000 spectrophotometer (Ozyme, Saint-Cyr-l’École, France) to warrant the lack of contaminants and a quantity of total RNA larger than 50 ng/µL. Then, 250 ng was reverse-transcribed into cDNA by Transcriptor First Stand cDNA Synthesis kit (04897030001, Roche Diagnostics) as recommended by the manufacturer with the following conditions: preparation (65 °C, 10 min with 60 µM random hexamer primer); cDNA synthesis (25 °C, 10 min; 55 °C, 30 min; 85 °C, 5 min; 4 °C on hold) in a final volume of 20 µL. Quantitative PCR reactions were performed in duplicate with fast Start Universal Probe Master kit and UPL-probe system (04914058001, Roche Diagnostics) in a LightCycler 480 Instrument (Roche Diagnostics) as recommended by the manufacturer with the following program for 12.5 pg of cDNA in a total volume of 12 µL: pre-incubation (95 °C, 10 min); 40 cycles of amplification (95 °C, 10 s; 60 °C, 30 s; 72 °C, 1 s); cooling (40 °C, 30 s). Specific primers (Eurogentec) were: CK5 forward 5′-TTCATGAAGATGTTCTTTGATGC-3′, reverse 5′-AGGTTGCGGTTGTTGTCC-3′ (amplicon length = 95 nt; probe #55); FOXJ1 forward 5′-CAGATCCCACCTGGCAGA-3′, reverse 5′-CGTACTGGGGGTCAATGC-3′ (amplicon length = 123 nt; probe #7); MUC5AC forward 5′-CACGTCCCCTTCAATATCCA-3′, reverse 5′-GGCCCAGGTCTCACCTTT-3′ (amplicon length = 91 nt; probe #33); MCIDAS forward 5′-CATCTGCCCCAACAGAATG-3′, reverse 5′-GATCCTCGTACACCGACACC (amplicon length = 140 nt; probe #55); HEATR2 forward 5′-ATCCTGTCCACCGTGCTG-3′, reverse 5′-CCAGGATGTCCTTTGTCACC-3′ (amplicon length = 94 nt; probe #33); RFX2 forward 5′-CTCAACCGCGTGGACTTT-3′, reverse 5′-CACACTCTCCTCGCACTGG-3′ (amplicon length = 69 nt; probe #68). Results for all expression data regarding transcripts were normalized to the expression of the house-keeping gene GAPDH amplified with the following primers: forward 5′-ACCAGGTGGTCTCCTCTGAC-3′, reverse 5′-TGCTGTAGCCAAATTCGTTG-3′ (amplicon length = 129 nt; probe #25). Relative gene expression was assessed by the ΔΔCt method [30] and expressed as fold change (log2, AB5E1 vs. control) when indicated.

### 2.7. Statistical Analysis

Quantitative variables were described with whisker plots as median; interquartile range and clinical parameters were represented with dot plot and median. Qualitative variables were compared using Fisher exact test. According to a limited number of patients, non-parametric Mann–Whitney and Kruskall–Wallis tests were used as appropriate. In all exploratory analyses, results with two-sided *p*-value < 0.05 were considered significant. The XLSTAT software (version 2019.1.3, Addinsoft, Paris, France) was used to analyze and reformat data within Excel for statistical analysis and represented with Prism (version 8, GraphPad software, San Diego, CA, USA).

## 3. Results

Thirty-two patients were included in the study: 17 COPD patients and 15 non-COPD patients (73% current or ex-smokers). Among COPD patients, the whole spectrum of spirometric severity was represented with a mean forced expiratory volume in one second (FEV_1_) of 54% (predicted value). The main characteristics of the patients are detailed in Table 1. Isolated AEC from the bronchial brushes were used to measure the CBF and establish ALI cultures.

**Figure 1:**
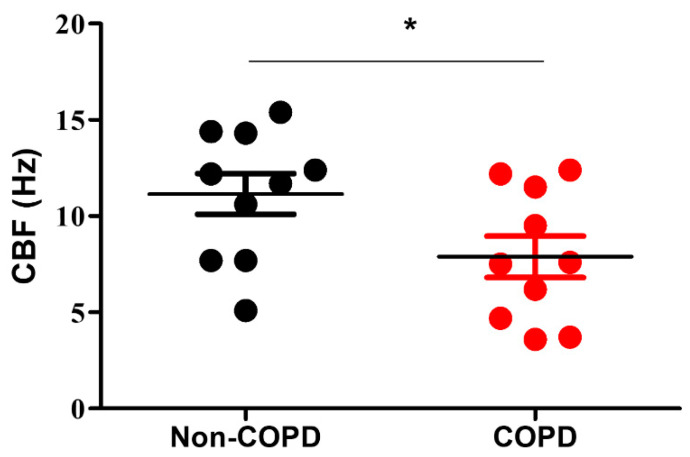
Cilia beat frequency of multiciliated cells is decreased in COPD patients. Dot plot (mean +/− SD) showing the CBF in non-COPD (black, *n* = 10) and COPD (red, *n* = 10) AEC. *, *p* < 0.05 non-COPD vs. COPD.

Exploring CBF on isolated airway epithelial cells (AEC) from bronchial brushes, we first confirmed the alteration of CBF in COPD patients. We measured a significant 30% decrease of CBF (11.15 +/− 3.37 Hz in non-COPD patients vs. 7.89 +/− 3.39 in COPD patients, *p* < 0.05) (Figure 1 and Supplemental Video S1 and S2).

**Figure 2:**
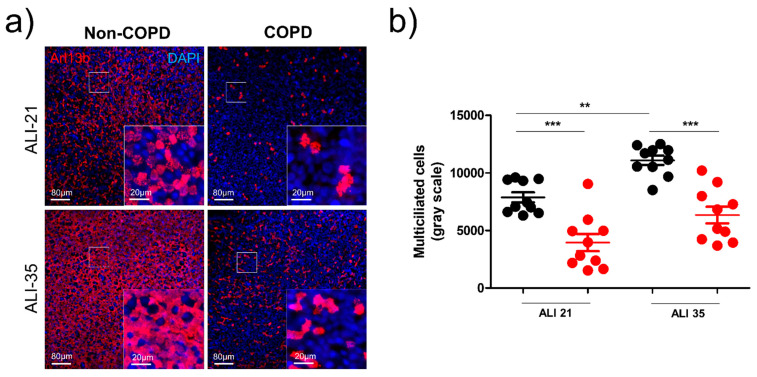
Multiciliated cell generation is decreased in COPD patients. (**a**) Examples of micrographs taken from AEC cultures from non-COPD and COPD patients at ALI-21 and ALI-35 showing motile cilia (Arl13b, red). Nuclei are stained in blue (DAPI). (**b**) Dot plot (mean +/− SD) represents the mean grey values of cilia-associated fluorescence at ALI-21 and ALI-35 in non-COPD AEC-derived ALI cultures (black, *n* = 10) and COPD AEC-derived ALI cultures (red, *n* = 10). ** *p* < 0.01, *** *p* < 0.001 non-COPD vs. COPD.

Then, we investigated multiciliated cell (MCC) formation during differentiation in AEC ALI cultures. We observed a significant two-fold decrease of MCC at ALI-21 in COPD patients (7869 +/− 1395 mean grey pixel density in non-COPD patients vs. 3939 +/− 2351 mean grey pixel density in COPD patients, *p* < 0.001) which was maintained at the end of differentiation at ALI-35 (11,085 +/− 1283 mean grey pixel density in non-COPD patients vs. 6335 +/− 2299 mean grey pixel density in COPD patients, *p* < 0.001) (Figure 2a,b). At a transcriptional level, the number of basal cells (CK5-expressing cells), mucous-secreting cells (MUC5AC-expressing cells), and MCCs (FOXJ1-expressing cells) were not significantly different in the early and late steps of AEC differentiation (Supplemental Figure S2).

**Figure 3:**
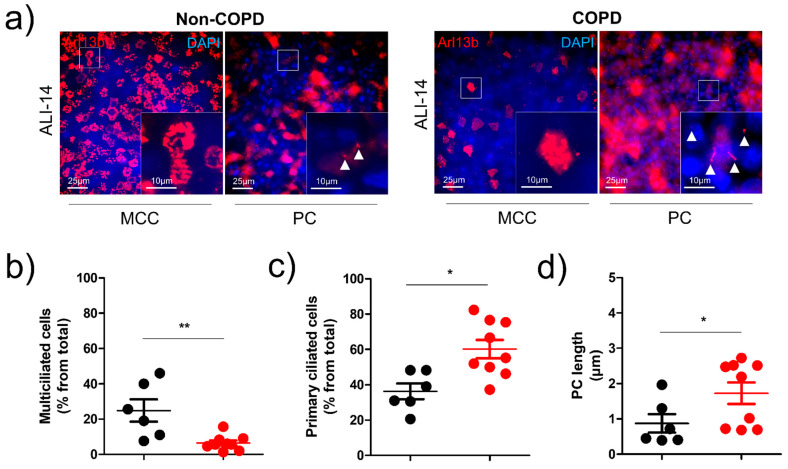
Primary ciliogenesis is altered in COPD-derived AEC ALI cultures. (**a**) Examples of micrographs taken from AEC cultures from non-COPD and COPD patients at ALI-14 showing motile cilia and primary cilia (Arl13b, red). Nuclei are stained in blue (DAPI). Magnification corresponding to the selected area is shown. White arrowheads indicate primary cilia. Dot plot (mean +/− SD) represents the percentage of MCC (**b**), the percentage of primary ciliated cells (**c**), and the length of PC (**d**) at ALI-14 in non-COPD AEC-derived ALI cultures (black, *n* = 6) and COPD AEC-derived ALI cultures (red, *n* = 8). * *p* < 0.05, ** *p* < 0.01 non-COPD vs. COPD.

We next assessed primary ciliogenesis. Since primary ciliogenesis precedes motile ciliogenesis during AEC differentiation, we focused here at an earlier in vitro time point. Motile ciliogenesis was also significantly decreased at ALI-14 in COPD patients (24.89 +/− 15.48% of MCC in non-COPD patients vs. 6.48 +/− 4.26% of MCC in COPD patients, *p* < 0.01) (Figure 3a,b). Interestingly, we observed an increase of PC (36.33 +/− 10.96% of cells with a PC in non-COPD patients vs. 60.22 +/− 15.58% in COPD patients, *p* < 0.05) (Figure 3c) which was associated with an increase of PC length (0.87 +/− 0.63 µm in non-COPD patients vs. 1.72 +/− 0.91µm in COPD patients, *p* < 0.05) (Figure 3d). At a transcriptional level, specific PCC markers responsible for the induction of multiciliogenesis including MCIDAS, HEATR2, and RFX2 were downregulated in COPD patients (Supplemental Figure S3). There was a significant 40% decrease of MCIDAS transcripts at ALI-7 (*p* < 0.05), a 60% decrease of HEATR2 transcripts at ALI-35 (*p* < 0.05), and a 65% decrease of RFX2 at ALI-35 (*p* < 0.05).

## 4. Discussion

Although CBF was analyzed in the context of COPD in animal models [31] or nasal brushing [5,6,7], it was only evaluated on ALI cultures of cells obtained from bronchial sampling [8], and no differences were observed between COPD-derived and non-COPD-derived cells. This prompted us to analyze CBF on bronchial AEC directly after bronchoscopy. In this condition, CBF was found to decrease suggesting that MCC may regain physiological cilia movements after repair. Nonetheless, we demonstrated that the cilia alteration persisted in COPD-derived respiratory epithelia: fewer MCC were produced while PC were more predominant and longer. This observation was consistent with our original assessment of PC alteration in COPD patients on lung tissues [11] and sustained a biological model placing the cilium at the core of cell cycle regulation and cell fate determinism [32]. Primary ciliogenesis is a necessary step during the cell cycle to orchestrate the fate of the two daughter cells: non-differentiated cells (able to form a PC) and differentiated cells (either MCC or secretory cells). Primary cilia are transient in homeostasis but their dysregulation (loss or maintenance) may induce an alteration of cell fate as we recently evidenced during AEC differentiation [27]. In COPD, the increase of PCC suggested that non-differentiated cells did not progress through the cell cycle and were blocked in G0. As a consequence, fewer non-differentiated cells initiated differentiation ultimately leading to a decrease in MCC, suggesting a global alteration of the differentiation programming. Experimental studies aiming to decipher the molecular mechanisms involved in the alteration of cilia in COPD patients are paramount to fully elucidate epithelial remodeling. This line of investigation may pave the way toward the clinical use of cilia and their abnormalities as diagnostic markers in a different spectrum of respiratory diseases such as COPD. It may also lead to considering new therapeutic pathways targeting cilium in COPD.

The cilium is a complex organelle exerting its function with molecular machinery that may only be resolved with electron microscopy. Recently, super-resolution microscopy coupled with focused ion beam scanning electron microscopy (FIB-SEM) demonstrated the presence of a hybrid cilium in MCC [33]. Exploring the structural abnormalities of all types of cilia in the context of respiratory diseases may contribute to the identification of new biomarkers. Additionally, the molecular and cellular analysis of cilia concomitant to smoke/pollutants exposure with additional technical approaches will help characterize and standardize diagnostics based on cilia dysregulation as it is routinely performed in the context of primary ciliary dyskinesia [23,34,35,36].

Since evaluating the structure and function of cilia (primary and motile) is crucial to understanding COPD pathogenesis given their involvement in mucociliary clearance, standardized investigations are required to establish guidelines in the evaluation of cilia and their alterations as diagnostic markers.

## Figures and Tables

**Table 1:** Baseline characteristics of the population.

	Non-COPD (*n* = 15)	COPD (*n* = 17)	*p*-Value
Sex ratio H/F	6/9	10/7	ns
Age (years)	52 ± 15	60 ± 12	ns
Smoking history	-	-	<0.01
Never smokers	4 (26%)	0	<0.05
Current-smokers	7 (47%)	5 (29%)	ns
Former-smokers	4 (26%)	12 (71%)	<0.05
Pack-years	21 ± 25	41 ± 24	<0.05
Spirometry	-	-	-
FEV_1_, % of predicted value	100 ± 17	54 ± 29	<0.0001
FVC, % of predicted value	102 ± 18	80 ± 22	<0.05
FEV_1_/FVC %	82 ± 10	48 ± 14	<0.0001
Spirometric GOLD 1/2/3/4	NA	4/3/6/4	-
GOLD A/B/C/D	NA	4/4/4/5	-
GOLD CAT	NA	3/4/3/7	-
Inhaled treatments	-	-	-
LABA	NA	12 (71%)	-
LAMA	NA	7 (41%)	-
ICS	NA	8 (47%)	-
Frequent exacerbation (>1/year)	-	7 (41%)	-
Respiratory symptoms	-	-	-
Dyspnea (mMRC) 0/1/2/3/4	4/7/2/2/0	4/4/5/3/1	ns
Cough	13 (86%)	16 (94%)	ns
Sputum	6 (40%)	13 (76%)	ns
Chronic bronchitis	4 (27%)	8 (47%)	ns

Data are expressed as mean ± SD or number (%); FEV1: forced expiratory volume in one second; FVC: forced vital capacity LABA: long-acting β2-agonist; LAMA: long-acting muscarinic-antagonist; ICS: inhaled corticosteroid; ns: non-significate.

## Data Availability

The data presented in this study are available on request from the corresponding author.

## Supplementary Materials

The following are available online at https://www.mdpi.com/article/10.3390/diagnostics11091579/s1, Figure S1: Technical workflow for CBF analysis on AEC, Figure S2: Transcript levels of the main differentiation markers in non-COPD and COPD AEC-derived ALI cultures, Figure S3: Transcript levels of the main motile ciliogenesis markers in non-COPD and COPD AEC-derived ALI cultures, Video S1: Example of a videomicroscope acquisition of a multiciliated cell from a non-COPD patient, Video S2: Example of a videomicroscope acquisition of a multiciliated cell from a COPD patient.

## Abbreviations

AEC	Airway epithelial cell
ALI	Air-liquid interface
CBF	Ciliary beat frequency
COPD	Chronic Obstructive Pulmonary Disease
FEV1	Forced Expiratory Volume in One second
MCC	Multiciliated cell
PC	Primary cilia
PCC	Primaryciliated cell
