# Supplementary material for: Impaired Ciliary Beat Frequency and Ciliogenesis Alteration during Airway Epithelial Cell Differentiation in COPD
Source: Diagnostics (Basel). 2021 Aug 31;11(9):1579. doi: 10.3390/diagnostics11091579 (PMC8469815; doi:10.3390/diagnostics11091579)
Supplement: Supplementary file 1 [file diagnostics-11-01579-s001.zip › diagnostics-1256542 supplementary file conversion.pdf]

diagnostics

MDPI

Supplementary File

Impaired Ciliary Beat Frequency and Ciliogenesis Alteration During Airway Epithelial Cell Differentiation in COPD

Julien Ancel [1,2], Randa Belgacemi [1], Zania Diabasana [1], Jeanne-Marie Perotin [1,2], Arnaud Bonnomet [1,3], Maxime Dewolf [2], Claire Launois [2], Pauline Mulette [1,2], Gaëtan Deslée [1,2], Myriam Polette [1,4] and Valérian Dormoy [1,*]

1 Inserm UMR-S1250, P3Cell, Université de Reims Champagne Ardenne, SFR CAP-SANTE, 51092 Reims, France; jancel@chu-reims.fr (J.A.); randa.belgacemi@lundquist.org (R.B.); zania.diabasana@inserm.fr (Z.D.); jmperotin-collard@chu-reims.fr (J.-M.P.); arnaud.bonnomet@univ-reims.fr (A.B.); pmulette@chu-reims.fr (P.M.); gdeslee@chu-reims.fr (G.D.); myriam.polette@univ-reims.fr (M.P.)
2 Department of respiratory diseases, Centre Hospitalier Universitaire de Reims, Hôpital Maison Blanche, 51092 Reims, France; mdewolf@chu-reims.fr (M.D.); claunois@chu-reims.fr (C.L.)
3 Platform of Cellular and Tissular Imaging (PICT), Université de Reims Champagne Ardenne, 51097 Reims, France
4 Department of biopathology, Centre Hospitalier Universitaire de Reims, Hôpital Maison Blanche, 51092 Reims, France
* Correspondence: valerian.dormoy@univ-reims.fr

Citation: Ancel, J.; Belgacemi, R.; Diabasana, Z.; Perotin, J.-M.; Bonnomet, A.; Maxime Dewolf; Launois, C.; Mulette, P.; Deslée, G.; Polette, M.; et al. Impaired Ciliary Beat Frequency and Ciliogenesis Alteration During Airway Epithelial Cell Differentiation in COPD. *Diagnostics* **2021**, *11*, 1579. https://doi.org/10.3390/diagnostics11091579

Academic Editor: Philippe A. Grenier

Received: 27 May 2021
Accepted: 27 August 2021
Published: 31 August 2021

Publisher's Note: MDPI stays neutral with regard to jurisdictional claims in published maps and institutional affiliations.

Copyright: © 2021 by the authors. Licensee MDPI, Basel, Switzerland. This article is an open access article distributed under the terms and conditions of the Creative Commons Attribution (CC BY) license (http://creativecommons.org/licenses/by/4.0/).

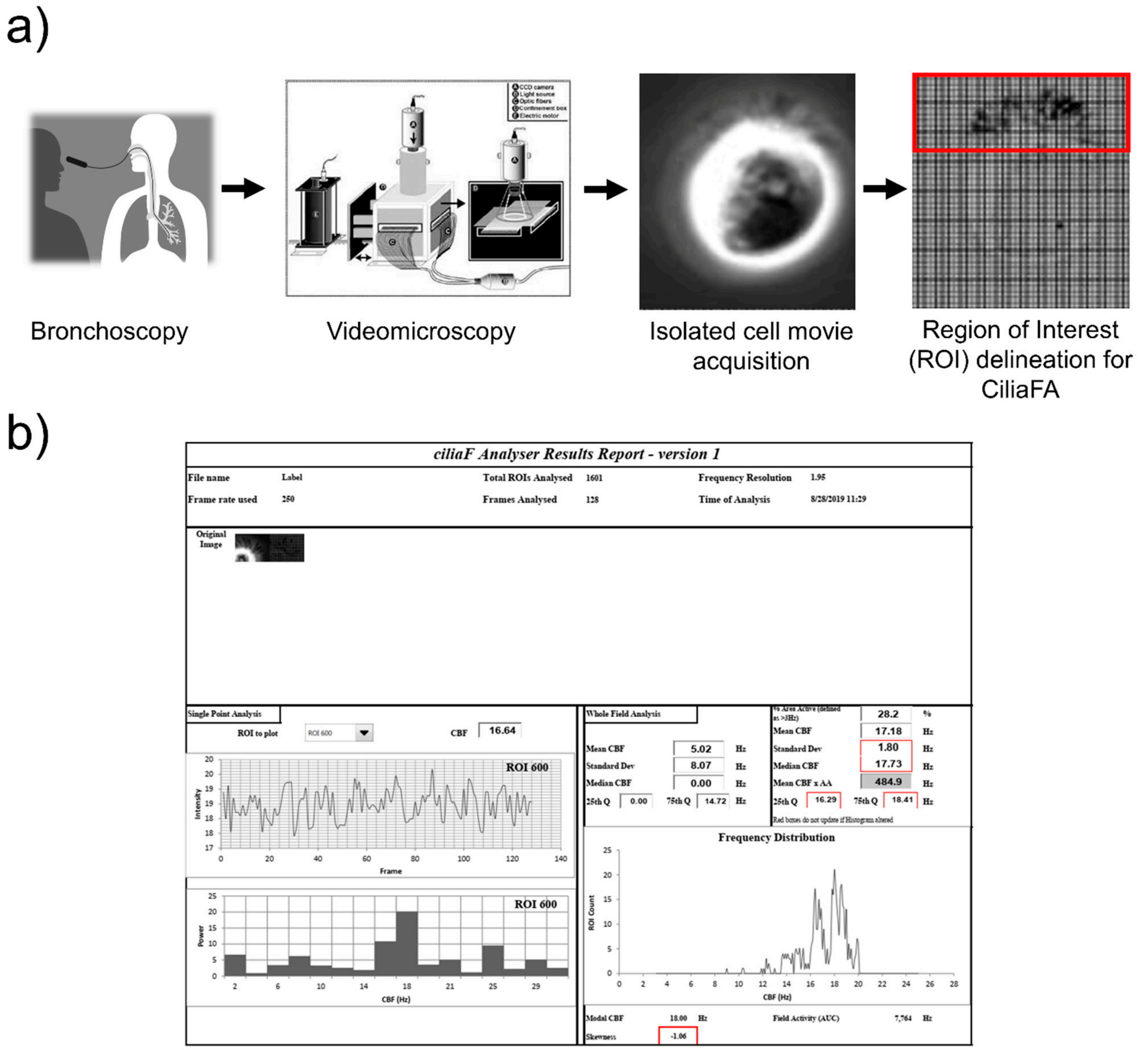

Figure S1. Technical workflow for CBF analysis on AEC. (**a**) Schema picturing the main steps to analyse CBF from clinical sampling to data analysis. (**b**) Example of a report obtained with CiliaF Analyser to measure CBF.

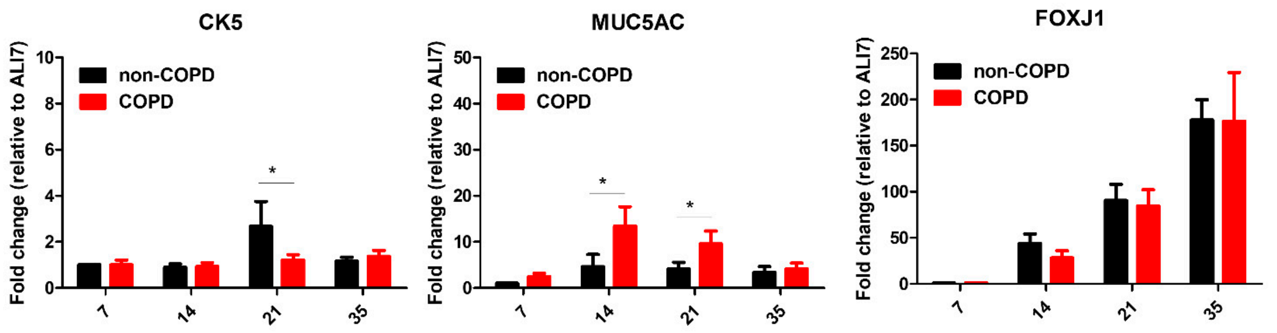

Figure S2. Transcript levels of the main differentiation markers in non-COPD and COPD AEC-derived ALI cultures. Histograms representing the relative mRNAs levels normalized to GAPDH obtained during the course of ALI cultures by RT-qPCR (n = 8) for differentiation markers (CK5, non-differentiated cells; MUC5AC, mucous-secreting cells; FOXJ1, ciliated cells). Means ± SEM of $2^{-\Delta\Delta Ct}$ are shown for each ALI time point.

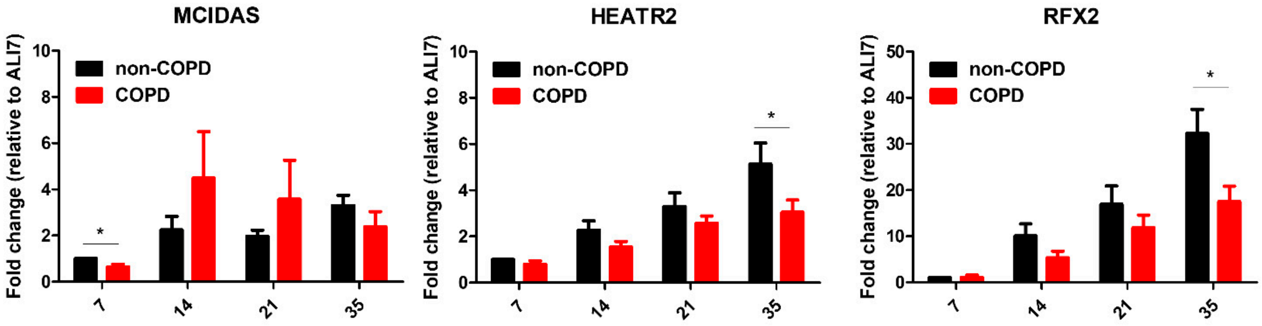

Figure S3. Transcript levels of the main motile ciliogenesis markers in non-COPD and COPD AEC-derived ALI cultures. Histograms representing the relative mRNAs levels normalized to GAPDH obtained during the course of ALI cultures by RT-qPCR (*n* = 8) for MCIDA, HEATR2, and RFX2. Means ± SEM of $2^{-\Delta\Delta Ct}$ are shown for each ALI time point.

Supporting Information Movies

Movie S1. Example of a videomicroscope acquisition of a multiciliated cell from a non-COPD patient.

The video was converted to 25 frames/sec.

Movie S2. Example of a videomicroscope acquisition of a multiciliated cell from a COPD patient.

The video was converted to 25 frames/sec.
